# Pristimerin, a triterpene that inhibits monoacylglycerol lipase activity, prevents the development of paclitaxel-induced allodynia in mice

**DOI:** 10.3389/fphar.2022.944502

**Published:** 2022-08-09

**Authors:** Altaf Al-Romaiyan, Willias Masocha

**Affiliations:** Department of Pharmacology and Therapeutics, Faculty of Pharmacy, Kuwait University, Safat, Kuwait

**Keywords:** triterpenes, monoacylglycerol lipase activity, chemotherapy-induced neuropathic pain, paclitaxel, mechanical allodynia, brain, paw skin

## Abstract

**Background:** Triterpenes such as euphol and pristimerin, which are plant secondary metabolites, were the first to be characterized as monoacylglycerol lipase (MAGL) inhibitors. MAGL inhibitors alleviate chemotherapy-induced neuropathic pain (CINP) in rodent models. Pristimerin has been shown to have additive anticancer activity with paclitaxel, a chemotherapeutic drug. However, the activity of pristimerin on CINP has not been evaluated.

**Objectives:** The aims of this study were to evaluate whether various triterpenes had activity against recombinant human MAGL and MAGL activity in mouse tissues, and whether pristimerin could prevent development of paclitaxel-induced mechanical allodynia.

**Methods:** The effects of four triterpenes betulinic acid, cucurbitacin B, euphol, and pristimerin on the activity human recombinant MAGL and MAGL activity of mice brain and paw skin tissues were evaluated using MAGL inhibitor screening and MAGL activity assay kits. The effects of treatment of female BALB/c mice with pristimerin intraperitoneally on the development of paclitaxel-induced mechanical allodynia were assessed using the dynamic plantar aesthesiometer and on nuclear factor-2 erythroid related factor-2 (*Nrf2*) gene expression in the paw skin were evaluated by real time polymerase chain reaction.

**Results:** Pristimerin inhibited the human recombinant MAGL activity in a concentration-dependent manner like JZL-195, a MAGL inhibitor. Betulinic acid, cucurbitacin B and euphol inhibited human recombinant MAGL activity but their effects were not concentration dependent and were less to that of pristimerin. Pristimerin inhibited both mouse brain and paw skin MAGL activity in a concentration-dependent manner. Paclitaxel induced mechanical allodynia and increase in MAGL activity in the paw skin. Treatment with pristimerin prevented the development of paclitaxel-induced mechanical allodynia and the paclitaxel-induced increase in MAGL activity. Pristimerin significantly upregulated mRNA expression of *Nrf2,* a regulator of endogenous antioxidant defense.

**Conclusion:** These results indicate that triterpenes inhibit human recombinant MAGL activity with varying degrees. Pristimerin inhibits both mouse brain and paw skin MAGL activity in a concentration-dependent manner, prevents both the development of paclitaxel-induced mechanical allodynia and the associated increase in MAGL activity in the paw skin, and might protect against paclitaxel-induced oxidative stress. Co-treatment with pristimerin and paclitaxel could be useful in the treatment of cancer and prevention of CINP.

## Introduction

Triterpenes are plant secondary metabolites, which contain about 30 carbon atoms, that have been isolated as active constituents of various plants (Jager et al., 2009; Parmar et al., 2013). They are classified into two main groups: tetracyclic and the pentacyclic triterpenes. Tetracyclic triterpenes include oleandrin, euphol and cucurbitacins (Jager et al., 2009; Parmar et al., 2013), of which euphol has been shown to inhibit recombinant rat monoacylglycerol lipase (MAGL) activity (King et al., 2009). Monoacylglycerol lipase is an enzyme that degrades the endocannabinoid 2-arachidonoylglycerol (2-AG) through hydrolysis to arachidonic acid and glycerol (Dinh et al., 2002; Saario et al., 2004). Endocannabinoids such as 2-AG and anandamide have analgesic activity (Rodriguez de Fonseca et al., 2005; Guindon and Hohmann, 2009; Munawar et al., 2017). Pentacyclic triterpenes are the largest group with compounds such as α-amyrin, β-amyrin, betulin, betulinic acid, pristimerin, lupeol, oleanolic acid, ursolic acid, maslinic acid, uvaol, and erythrodiol (Jager et al., 2009; Parmar et al., 2013), of which α-amyrin, β-amyrin and pristimerin have been shown to inhibit MAGL activity (King et al., 2009; Chicca et al., 2012). Various triterpenes such as lupeol, tormentic acid, betulin, betulinic acid and epibetulin have been reported to have antinociceptive and antiallodynic activities in acute pain, inflammatory pain and neuropathic pain caused by partial constriction of the sciatic nerve (Calixto et al., 2000; Bortalanza et al., 2002; Parmar et al., 2013). However, the effects of some of these triterpenes on MAGL activity and on chemotherapy-induced neuropathic pain (CINP) have not yet been evaluated.

Paclitaxel, a chemotherapeutic drug used in the treatment of breast cancer and other solid tumors causes dose-limiting CINP referred to as paclitaxel-induced neuropathic pain (PINP) (Polomano and Bennett, 2001; Cata et al., 2006; Wolf et al., 2008). Unfortunately, there is a scarcity of drugs to effectively alleviate or prevent the development of PINP. Currently, only duloxetine has moderate recommendation for the treatment of CINP, including PINP, while other agents useful in other types of neuropathic pain may also be used because of limited options for treating CINP (Hershman et al., 2014). Therefore, further research into both the pathophysiology of CINP and the search of novel, more efficacious and safer agents for the treatment of CINP is essential.

Recent data from our laboratory show that there is a decrease in the levels of 2-AG in the paw skin of mice with paclitaxel-induced mechanical allodynia, without a change in the protein levels of the enzyme MAGL (Thomas et al., 2020). Administration of 2-AG or a MAGL inhibitor JZL184 into the right hind paw skin of mice alleviated paclitaxel-induced mechanical allodynia in the injected right paw but did not affect the uninjected left paw (Thomas et al., 2020). These findings suggest that MAGL inhibitors could be useful for management of CINP.

Pristimerin has been reported to have anticancer activities against various types of cancer cell lines including those of breast cancer, cervical cancer, prostate cancer, gliomas, and fibrosarcoma (Yang et al., 2008; Byun et al., 2009; Lee et al., 2013; Yan et al., 2013; Hayashi et al., 2020). Pristimerin has also been shown to have additive anticancer activity with paclitaxel against breast cancer cell lines (Lee et al., 2018). This could lead to lower doses of paclitaxel being used for treating cancer and thus reduce the occurrence of the dose dependent PINP. However, the effects of pristimerin on the development of PINP or any CINP have not been evaluated.

The objectives of this study were to evaluate whether various triterpenes, including pristimerin had activity against recombinant human MAGL and MAGL activity in mouse brain and paw skin tissue, and whether pristimerin could prevent the development of mechanical allodynia induced by paclitaxel in mice. Other objectives were to evaluate if treatment with paclitaxel affects MAGL activity in the periphery at a time when female mice develop paclitaxel-induced mechanical allodynia, and also whether administration of pristimerin could reduce/prevent the effect of paclitaxel on MAGL activity.

## Materials and methods

### Animals

Female BALB/c mice (8–12 weeks old; *n* = 54) were used in this study and were supplied by the Animal Resources Centre at the Health Sciences Centre (HSC), Kuwait University. In the current study, female mice were used following the recommendation of the members of the Sex, Gender and Pain Special Interest Group of the International Association for the Study of Pain (IASP) that “all pain researchers consider testing their hypotheses in both sexes, or if restricted by practical considerations, only in females” (Greenspan et al., 2007). The strain was chosen based on availability and our previous studies with the same strain (Parvathy and Masocha, 2013; Masocha and Thomas, 2019; Thomas et al., 2020). The animals were handled in compliance with Directive 2010/63/EU of the European Parliament and of the Council on the protection of animals used for scientific purposes. All animal experiments were approved by the Ethical Committee for the use of Laboratory Animals in Teaching and in Research, HSC, Kuwait University (Ref: 23/VDR/EC/, Date 5/8/2020). They were kept in temperature controlled (24 ± 1°C) rooms with food and water *ad libitum*. All experiments were performed between 0800–1600 h to reduce the effects of circadian variations in pharmacological effects.

### Evaluation of inhibitory potential of triterpenes on human monoacylglycerol lipase activity

The effects of four triterpenes, betulinic acid (Sigma–Aldrich, Germany), cucurbitacin B (Phytolab, Germany), euphol (TargetMol, United States) and pristimerin (Tocris, United Kingdom), on human recombinant MAGL activity was evaluated *in vitro* using the Monoacylglycerol lipase assay inhibitor screening colorimetry-based assay kit (Cayman Chemical, Ann Arbor, MI, United States) following the manufacturer’s instructions. MAGL hydrolyses the substrate (4-nitrophenyl acetate) and generates 4-nitrophenol, whose absorbance was taken at 405 nm on microplate reader. JZL195, a dual inhibitor fatty acid amide hydrolase (FAAH) and MAGL, was used as a MAGL inhibitor reference standard for the assay.

Briefly, different concentrations of betulinic acid (0.1, 1, 10, 100 and 200 μM), cucurbitacin B (0.1, 1, 10, 100 and 200 μM), euphol (0.001, 0.01, 0.1 and 1 μM), pristimerin (0.001, 0.01, 0.1 and 1 μM) and JZL195 (0.001, 0.01, 0.1, 1 and 4.4 μM) were prepared in a solution made up of 50% dimethylsulfoxide (DMSO; Sigma-Aldrich) and 50% assay buffer. A mixture of assay buffer (150 μl), diluted human recombinant MAGL (10 μl), and solvent or inhibitor (10 μl) at different concentrations was incubated for 15 min at 25°C in a 96 well microplate. The reaction was initiated by adding 10 µl of MAGL substrate to all the wells being used and the plate incubated at 25°C in the microplate reader. The plate was shaken for 10 s to mix every time before taking absorbance at 405 nm for various time points (5, 10, 30, and 60 min) using a microplate reader (SpectraMax^®^ iD3, Molecular Devices). The experiment was performed in triplicate.

%Activity was calculated as %Initial Activity = Inhibitor activity/Initial activity*100, following the manufacturer’s protocol. Initial activity is the absorbance in the wells the reaction was run without inhibitor.

### Evaluation of inhibitory potential of triterpenes on mouse brain and paw skin tissue monoacylglycerol lipase activity

The effects of pristimerin on mouse tissue MAGL activity was evaluated *in vitro* using the Monoacylglycerol Lipase (MAGL) Activity Assay Kit (Fluorometric) (ab273326; Abcam) following the manufacturer’s instructions. MAGL cleaves a fluorescent substrate to generate arachidonic acid and fluorescent metabolite and the increased fluorescence is measured at Ex/Em 360/460 nm. A MAGL inhibitor (name not disclosed by company because it is proprietary information) is also used to differentiate MAGL activity from other sources of fluorescence. Pristimerin was selected because it was the one with best activity in inhibiting human recombinant MAGL amongst the triterpenes tested. JZL195, a dual inhibitor of FAAH and MAGL, was used as a MAGL inhibitor reference standard for the assay.

Briefly, mouse brain and paw skin tissues were dissected out and homogenized, 10 mg of tissue in 100 μl MAGL assay buffer, centrifuged at 10,000 g for 15 min (4°C), and the supernatant was collected, protein concentrations determined by the by Bicinchoninic acid (BCA) assay using bovine serum albumin (BSA) as standard, The supernatants were aliquoted and stored at −80°C until the time to measure MAGL activity using the assay kit (Abcam) at around 0.04–0.05 mg per reaction. Different concentrations (0.01, 0.1, 1, 4.4 and 10 μM) of pristimerin and JZL195 were prepared in a solution made up of 50% DMSO and 50% assay buffer. A mixture of assay buffer (60–75 μl), mouse brain or paw skin supernatant (5 or 20 μl, respectively), and solvent or inhibitor (10 μl) at different concentrations was incubated for 30 min at 37°C in a 96 well black microplate. The reaction was initiated by adding 10 µl of MAGL substrate to all the wells being used and the plate incubated at 37°C in the microplate reader (SpectraMax^®^ iD3, Molecular Devices) set at low, 1 OD (optical density) and integration at 140 ms. The plate was shaken for 5 s to mix every time before measuring fluorescence at Ex/Em 360/460 in kinetic mode for 60 min reading every 10 min.

% MAGL activity was calculated as follows:
FS = Absorbance of well of interest—absorbance of well with vehicle plus kit inhibitor, following the manufacturer’s protocol.
% MAGL activity = FS of drug/FS of vehicle*100

### Administration of paclitaxel to induce mechanical allodynia

Paclitaxel (Tocris, United Kingdom) was dissolved in a solution made up of 50% Cremophor EL and 50% absolute ethanol to a concentration of 6 mg/ml and stored at − 20°C, for a maximum of 14 days, and diluted in normal saline (NaCl 0.9%), to a final concentration of 0.2 mg/ml just before administration. Paclitaxel 2 mg/kg was injected intraperitoneally (i.p.) in a volume of 10 ml/kg, once daily for 5 consecutive days as previously described (Parvathy and Masocha, 2013). The vehicle for pristimerin that consisted of 1.7% Cremophor EL and 1.7% ethanol in normal saline was injected to control animals. This treatment regimen produces mechanical allodynia in mice (Masocha and Thomas, 2019).

### Drug administration

**FIGURE 1:**
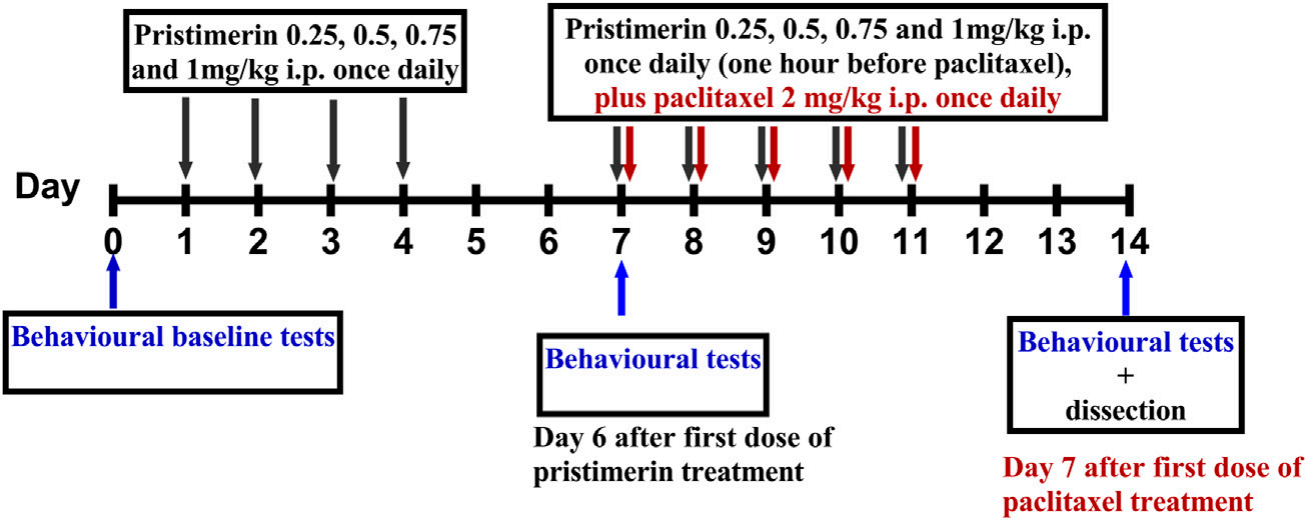
Drug administration schedule for treatment with pristimerin against paclitaxel-induced allodynia. The black arrows indicate the days pristimerin was administered, the maroon arrows indicate the days when paclitaxel was administered, while the blue arrows indicate when the behavioral tests were performed.

Pristimerin was dissolved in 100% DMSO to a concentration of 10 mg/ml and stored at −20°C and diluted in 2% DMSO in phosphate buffered saline (PBS) to a final concentration just before administration. Pristimerin 0.25, 0.5, 0.75, and 1 mg/kg was injected i.p. in a volume of 10 ml/kg, once daily for 4 consecutive days starting 6 days before the administration of paclitaxel. This was followed by administration of pristimerin 0.25, 0.5, 0.75 and 1 mg/kg for 5 days 1 h before paclitaxel injection (Figure 1). The vehicle for pristimerin that consisted of 3% DMSO in PBS was injected to control animals in the same pattern as pristimerin (Figure 1).

### Assessment of mechanical allodynia

Mechanical allodynia was measured using the dynamic plantar aesthesiometer (Ugo Basile, Italy). Briefly, mice were left to habituate for 30–60 min inside plastic enclosures on top of a perforated platform. A microprocessor, which was programmed to automatically lift a metal filament that exerted a linearly increasing force (0.25 g/s with cut-off time of 20 s) on the hind paw, was pressed to start when the mice were settled. A stop signal was automatically attained, either when the animal removed the paw or at the cut-off force of 5 g. Withdrawal thresholds in response to the mechanical stimulus were automatically recorded in grams. The hind paws were tested at least 3 times. The baseline mechanical thresholds were assessed before initiation of treatment with pristimerin and before the induction of the neuropathic pain with paclitaxel.

### Assessment of monoacylglycerol lipase activity in mice paw skin

Briefly, paw skin tissues were dissected out after treatment with pristimerin and paclitaxel (see Figure 1) on day 7 after paclitaxel treatment and homogenized, 10 mg of tissue in 100 μl MAGL assay buffer, centrifuged at 10,000 g for 15 min (4°C), and the supernatant was collected, protein concentrations determined by the by BCA assay using BSA as standard, The supernatants were aliquoted and stored at −80°C until the time to measure MAGL activity using the MAGL Activity Fluorometric assay kit (Abcam) at around 0.05 mg per reaction. A mixture of assay buffer (60 μl), paw skin supernatant (20 μl), and solvent or inhibitor (10 μl) at different concentrations was incubated for 30 min at 37°C in a 96 well black microplate. The reaction was initiated by adding 10 µl of MAGL substrate to all the wells being used and the plate incubated at 37°C in the microplate reader (SpectraMax^®^ iD3, Molecular Devices) set at low, 1 OD (optical density) and integration at 140 ms. The plate was shaken for 5 s to mix every time before measuring fluorescence at Ex/Em 360/460 in kinetic mode for 60 min reading every 10 min.

Relative fluorescence units (360/460 nm) of the MAGL-specific signal per mg of the samples was determined as follows: Firstly, the relative fluorescence units were corrected for weight of samples by dividing it by the amount of samples used in the reaction in mg. Secondly, the corrected relative fluorescence units of the wells with kit inhibitor were subtracted from that of well without inhibitor.

The area under the curve of the relative fluorescence units (360/460 nm) for each sample was calculated from zero to 60 min using GraphPad Prism 9.0.

### Real time RT-PCR

Expression of *Nrf2* mRNA was quantified relative to the expression of the house keeping gene *18s* (18S ribosomal RNA) using real time PCR. Total RNA was extracted from fresh frozen paw skins, reverse-transcribed into cDNA and real time PCR performed using QuantStudio™ 7 Flex Real-Time PCR System (Applied Biosystems) as described previously (Masocha, 2009). The primers for Nrf2 and 18s rRNA, were purchased from Sigma-Aldrich. The sequences of the primers used were: *Nrf2* forward, 5′- CAG​CAT​AGA​GCA​GGA​CAT​GGA​G-3′ and reverse, 5′-GAA​CAG​CGG​TAG​TAT​CAG​CCA​G-3′; *18s* forward, 5′-CGG​CTA​CCA​CAT​CCA​AGG​AA-3′ and reverse, 5′-GCT​GGA​ATT​ACC​GCG​GCT -3′. The threshold cycle (Ct) values for all cDNA samples were obtained and the level of mRNA for each sample were normalized to *18s* (housekeeping gene) ΔCt. The relative expression of the gene of interest was calculated using -2^−ΔΔCt^ method (Livak and Schmittgen, 2001).

### Statistical analyses

Data were tested for normality using the D’Agostino-Pearson normality test and if data passed the normality test parametric tests were used, however, if they failed the normality test non-parametric tests were used. Statistical analyses were performed using Student’s *t* test, Mann-Whitney test, one-way analysis of variance (ANOVA) followed by Tukey’s multiple comparisons test, Kruskal-Wallis test followed by Dunn’s multiple comparisons test, or two-way repeated measures ANOVA followed by Dunnett’s or Tukey’s multiple comparisons test using GraphPad Prism software (version 9.0). The differences were considered significant at *p* < 0.05. The results in the text and figures are expressed as the Mean ± SEM.

## Results

### Inhibitory effects of triterpenes on recombinant human monoacylglycerol lipase activity

**FIGURE 2:**
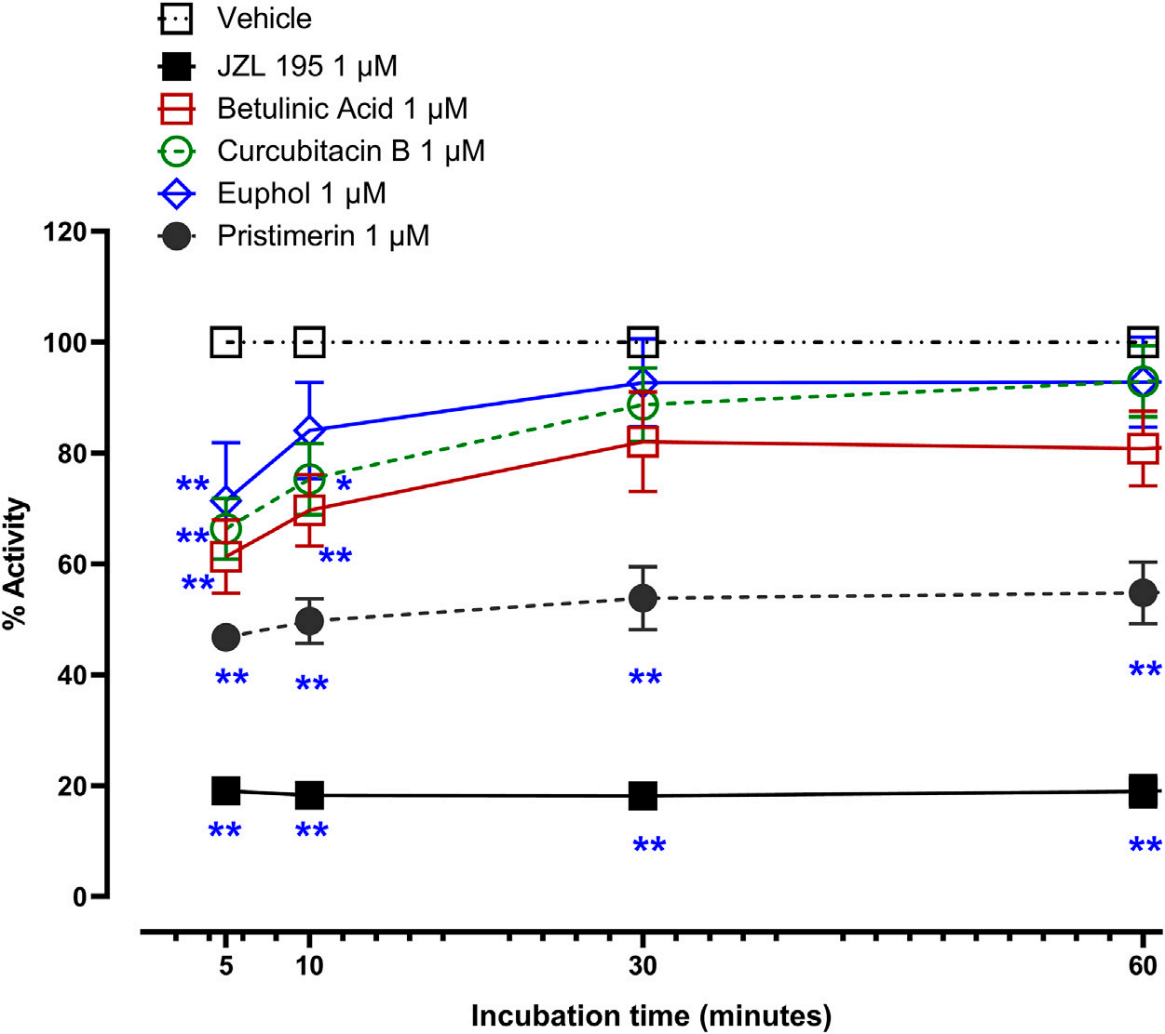
Inhibitory effects of 1 µM betulinic acid, cucurbitacin B, euphol, pristimerin and JZL195 on human recombinant MAGL activity over time. Each point represents the mean ± SEM of values obtained from 3 experiments. *p < 0.05, **p < 0.01 compared to drug vehicle at the same time after treatment (two-way repeated measures ANOVA followed by Dunnett’s multiple comparisons test).

At 1 µM JZL195 and the triterpenes significantly inhibited the activity of recombinant human MAGL (Figure 2, *p* < 0.05). Two-way repeated ANOVA showed there was a significant effect of treatment with JZL195 [F (1, 5) = 3094, *p* < 0.0001], betulinic acid [F (1, 5) = 15.54, *p* = 0.0109], cucurbitacin B [F (1, 5) = 13.49, *p* = 0.0144] and pristimerin [F (1, 5) = 243.2 *p* < 0.0001], but not euphol [F (1, 6) = 2.805, *p* = 0.1450], on the activity of recombinant human MAGL compared to vehicle. The magnitude of inhibition (calculated at 10 min of incubation) was JZL195 (82% inhibition) > pristimerin (50% inhibition) > betulinic acid (30% inhibition) ≥ cucurbitacin B (25% inhibition) > euphol (16% inhibition). The inhibition of JZL195 and pristimerin on MAGL activity was maintained from 5 till 60 min, when the experiment was terminated, whereas the other triterpenes lost some activity with time (Figure 2).

**FIGURE 3:**
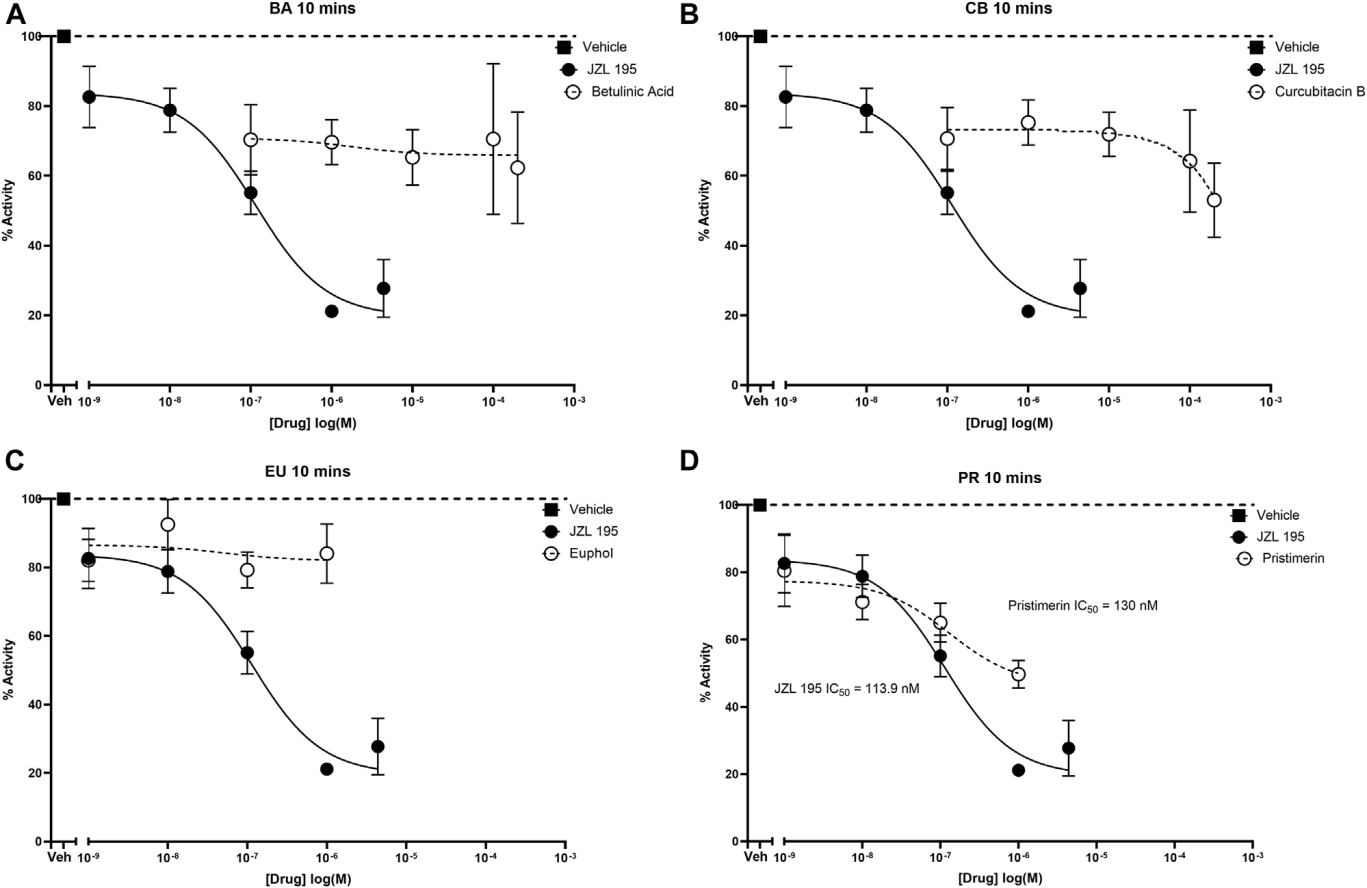
Inhibitory effects of various concentrations of betulinic acid, cucurbitacin B, euphol, pristimerin and JZL195 on human recombinant MAGL after 10 min of incubation. Each point represents the mean ± SEM of values obtained from 3 to 5 experiments.

JZL195 and pristimerin inhibited human recombinant MAGL activity in a concentration-dependent manner with median inhibitory concentrations (IC50s) of 113.9 and 130 nM, respectively (Figure 3D). Although betulinic acid, cucurbitacin B and euphol inhibited the activity of human recombinant MAGL their effects were not concentration-dependent, and thus could not calculate IC50 values (Figures 3A–C).

### Inhibitory effects of triterpenes on mouse brain and paw skin

**FIGURE 4:**
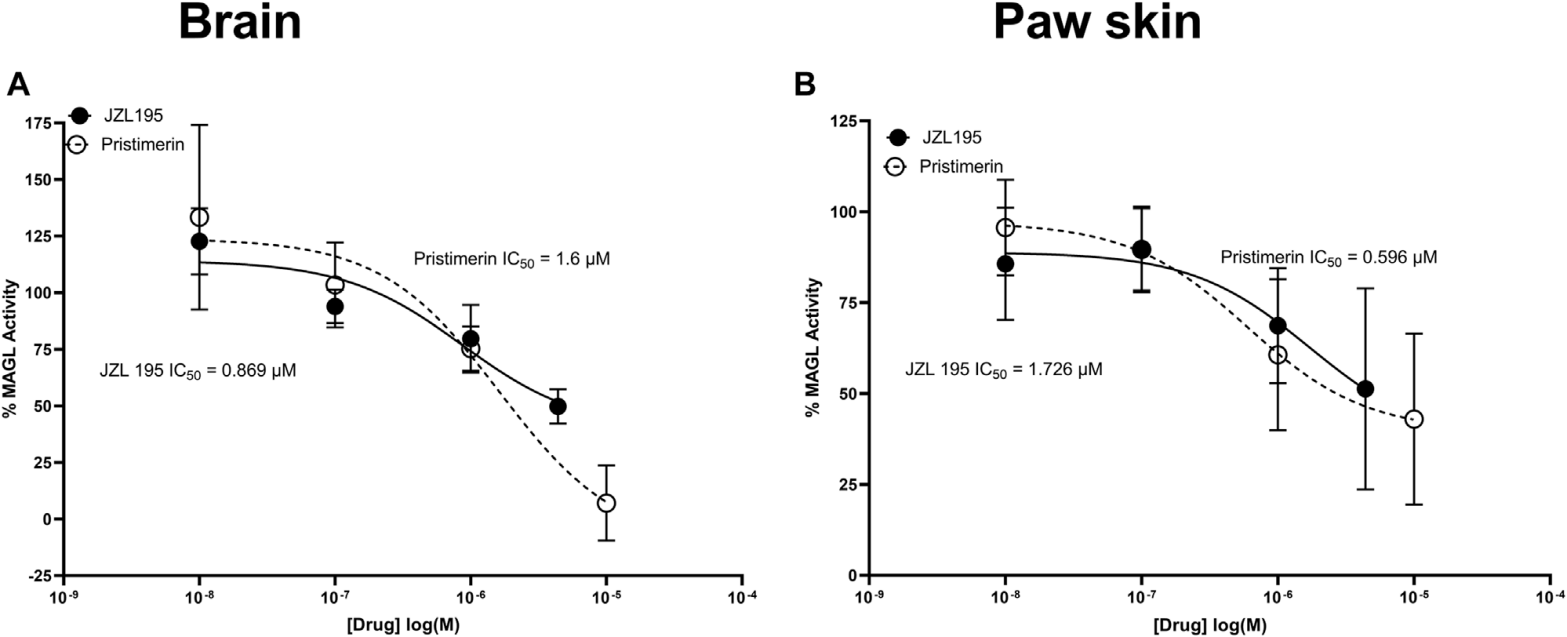
Inhibitory effects of various concentrations of pristimerin and JZL195 on mouse **(A)** brain and **(B)** paw skin MAGL after 10 min of incubation. Each point represents the mean ± SEM of values obtained from 3 experiments from samples pooled from 8 animals.

JZL195 and pristimerin inhibited both mouse brain and paw skin MAGL activity in a concentration-dependent manner with IC_50_s of 0.869 and 1.6 µM in the brain, respectively and 1.726 and 0.596 µM in the paw skin, respectively (Figure 4).

### Effects of pristimerin on the development of paclitaxel-induced mechanical allodynia in mice

**FIGURE 5:**
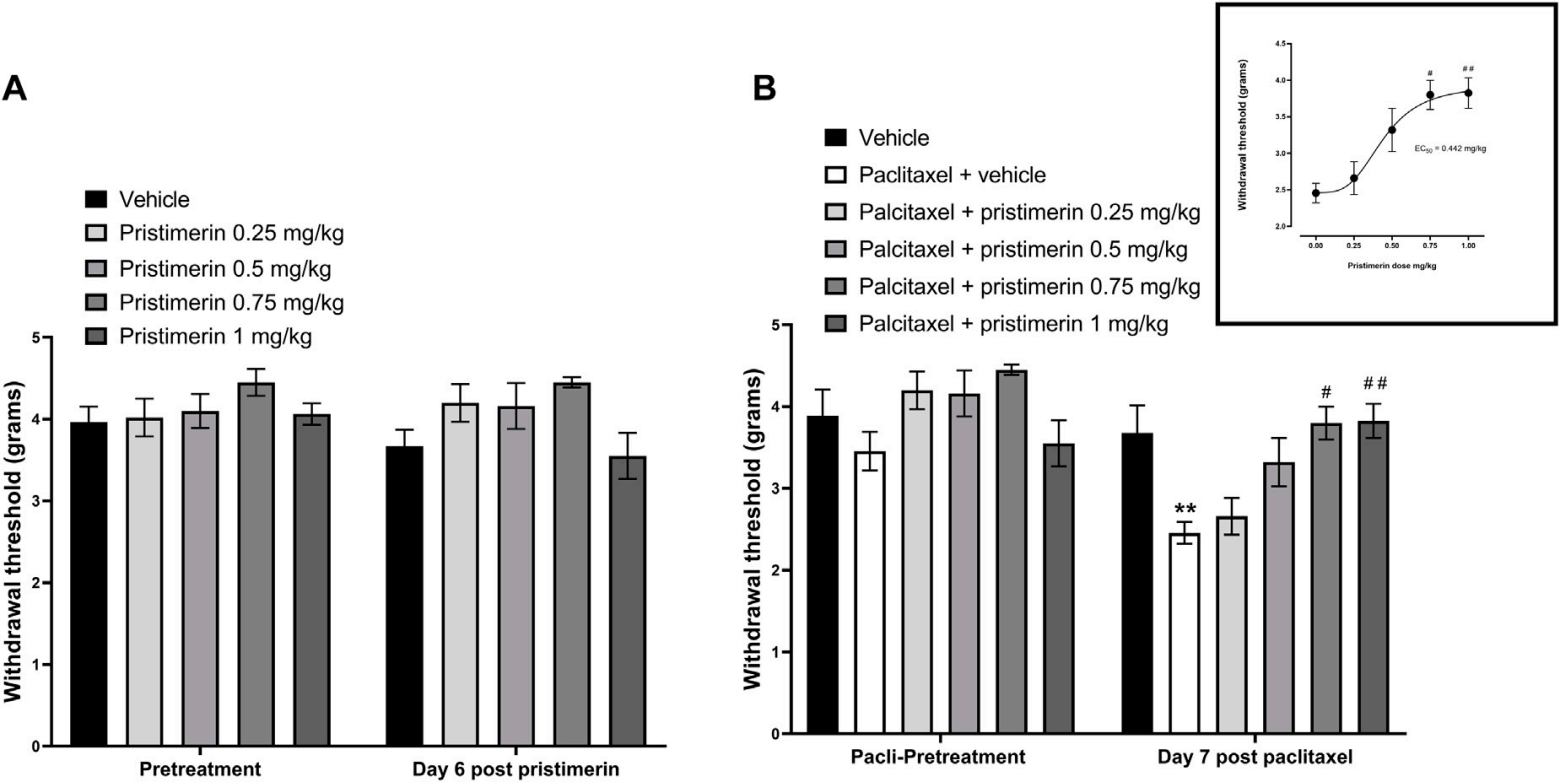
Pristimerin prevents the development of paclitaxel-induced mechanical allodynia in female BALB/c mice. **(A)** The effect of treatment of naïve mice with pristimerin on response to mechanical stimuli. Each bar represents the mean ± SEM of values obtained from four to eighteen animals. **(B)** The effect of treatment with pristimerin on the development of paclitaxel-induced mechanical allodynia at day 7 post-first injection (dpi) of paclitaxel. Each bar represents the mean ± SEM of values obtained from four to nine animals. **p < 0.01 compared to pretreatment values (Mann-Whitney test), and #*p* < 0.05, ##*p* < 0.01 compared to mice treated with paclitaxel + vehicle (two-way repeated measures ANOVA followed by Tukey’s multiple comparisons test and for the insert Kruskal-Wallis test followed by Dunn’s multiple comparisons test).

Two-way repeated ANOVA showed that treatment of naïve mice with pristimerin 0.25, 0.5, 0.75, and 1 mg/kg i.p. daily for 4 consecutive days did not cause any significant changes to the withdrawal threshold of mice to the dynamic plantar aesthesiometer on day 6 compared to pretreatment baseline values or vehicle treatment [Figure 5A, (4, 35) = 1.439, *p* = 0.2418].

Treatment with paclitaxel significantly reduced the withdrawal threshold of mice to the dynamic plantar aesthesiometer on day 7 compared to pretreatment values, with values of 2.46 ± 0.13 g versus 3.46 ± 0.24 g, respectively [t (16) = 3.691, *p* = 0.002], whereas vehicle only treated animals had similar values on day 7 compared to pretreatment values 3.68 ± 0.34 g versus 3.89 ± 0.32 g, respectively [t (16) = 0.4543, *p* = 0.6567]. On the other hand, mice treated with pristimerin 0.75 and 1 mg/kg plus paclitaxel had withdrawal threshold on day 7 similar to the pretreatment values, with values of 3.80 ± 0.20 g and 3.83 ± 0.21 g versus 4.45 ± 0.07 g and 3.55 ± 0.28 g, respectively [Figure 5B, U = 1.5, *p* = 0.0857, t (14) = 0.7815, *p* = 0.4475, respectively]. Kruskal-Wallis test showed that coadministration of pristimerin with paclitaxel significantly changed the withdrawal thresholds of mice compared to the paclitaxel plus vehicle group [H (5) = 18.75, *p* = 0.0009]. Post-hoc analysis showed that the lower doses of pristimerin 0.25 and 0.5 mg/kg did not significantly change withdrawal thresholds compared to paclitaxel plus vehicle-treated mice (Figure 5B, *p* > 0.05), whereas the higher doses 0.75 and 1 mg/kg were significantly higher than those of the mice treated with paclitaxel plus vehicle (Figure 5B, *p* < 0.05). Treatment with pristimerin produced a dose-dependent protection against the development of paclitaxel-induced mechanical allodynia with an ED_50_ of 0.442 mg/kg (Figure 5B).

### Effects of treatment with pristimerin on paclitaxel-induced monoacylglycerol lipase activity in the paw skin

**FIGURE 6:**
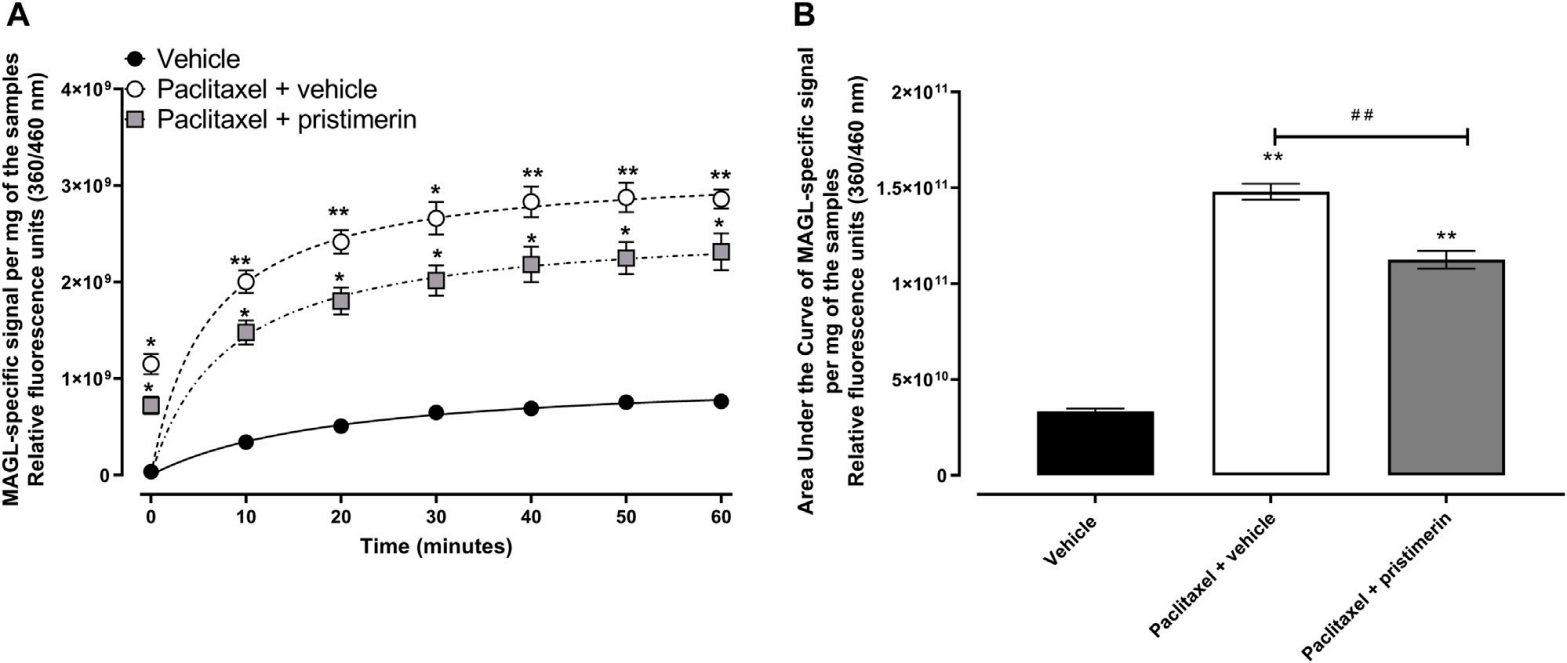
Pristimerin prevents the paclitaxel-induced increase in MAGL activity in the paw skin of female BALB/c mice. **(A)** Relative fluorescence units (360/460 nm) over time as a product of MAGL-specific signal per mg of the paw skin samples from vehicle-, paclitaxel plus vehicle- and paclitaxel plus pristimerin-treated mice at day 7 post-first injection (dpi) of paclitaxel. Each symbol represents the mean ± SEM of values obtained from 3 experiments from samples pooled from 4 animals. *p < 0.05, **p < 0.01 compared to mice treated with vehicle only (two-way repeated measures ANOVA followed by Tukey’s multiple comparisons test). **(B)** Area under the curve of relative fluorescence of MAGL-specific signal per mg of the paw skin samples from vehicle-, paclitaxel plus vehicle- and paclitaxel plus pristimerin-treated mice at day 7 dpi of paclitaxel. Each symbol represents the mean ± SEM of values obtained from 3 experiments from samples pooled from 4 animals. **p < 0.01 compared to mice treated with vehicle only and ##*p* < 0.01 compared to mice treated with paclitaxel + vehicle (one-way ANOVA followed by Tukey’s multiple comparisons test).

Treatment with paclitaxel significantly increased the MAGL activity in the paw skin compared to vehicle-only-treated control mice (Figures 6A,B, *p* < 0.05). Two-way repeated ANOVA showed there was a significant effect of treatment with paclitaxel [F (1, 4) = 194.6, *p* = 0.0002] and paclitaxel plus pristimerin [F (1, 4) = 74.61, *p* = 0.0010] on the activity of MAGL compared to treatment with vehicle. Two-way repeated ANOVA showed there was a significant effect of treatment with pristimerin [F (1, 4) = 8.578, *p* = 0.0429] on the activity of MAGL from paw skins of mice with paclitaxel-induced allodynia compared to treatment with vehicle. The relative fluorescence units (360/460 nm) of the MAGL-specific signal per mg of the paw skin samples and AUC were higher in paclitaxel-treated mice compared to vehicle-only-treated control mice (Figures 6A,B, *p* < 0.05). The AUC of relative fluorescence units was 1.48 × 10^11^ for paclitaxel-treated mice versus 3.35 × 10^10^ for vehicle-treated mice (*p* < 0.05).

One-way ANOVA showed that there were differences between treatment groups [F (2, 6) = 252.9, *p* < 0.0001]. Treatment with pristimerin significantly reduced the paclitaxel-induced increase in MAGL activity in the paw skin, i.e., the AUC of the relative fluorescence of the MAGL-specific signal per mg of the paw skin samples of mice treated with paclitaxel plus pristimerin were significantly lower than those of mice treated with paclitaxel plus vehicle (Figure 6B, *p* < 0.01), although still higher than the vehicle-only-treated control mice (Figure 6B, *p* < 0.01). The AUC of relative fluorescence units was 1.12 × 10^11^ for paclitaxel plus pristimerin-treated mice versus 1.48 × 10^11^ for paclitaxel plus vehicle-treated mice (*p* < 0.05).

### Effects of treatment with pristimerin and paclitaxel on nuclear factor-2 erythroid related factor-2 gene expression in the paw skin

**FIGURE 7:**
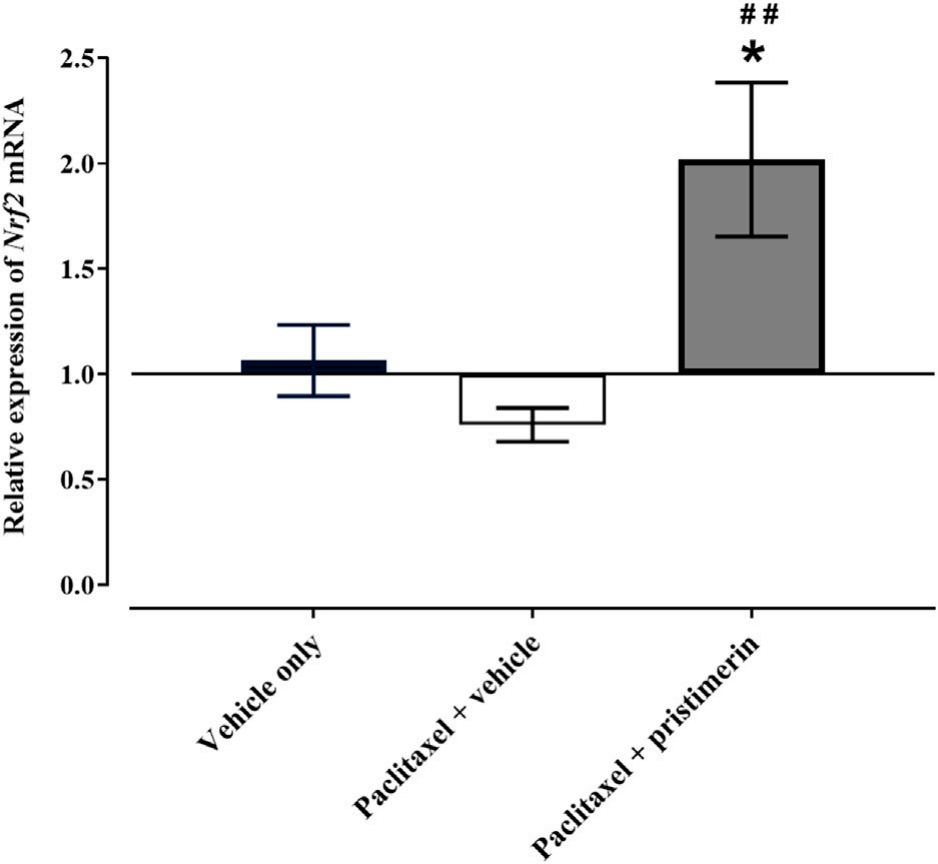
Effect of treatment with paclitaxel or paclitaxel + pristimerin on the relative expression of *Nrf2* mRNA in paw skins of female BALB/c mice at day 7 post first paclitaxel dose. Each bar represents the mean ± SEM of values obtained from 5-6 animals. *p < 0.05 compared to control mice (treated with vehicle only) at day 7 and ##*p* < 0.01 compared to mice treated with paclitaxel + vehicle (one-way ANOVA was followed by Tukey’s multiple comparisons test).

One-way ANOVA showed that there were differences between treatment groups in the *Nrf2* gene expression in the paw skin [F (2, 13) = 7.741, *p* = 0.0061]. Post-hoc analysis showed that treatment with paclitaxel did not significantly change the expression of *Nrf2* mRNA in the paw skin (Figure 7, *p* > 0.05). However, there was a trend towards reduction compared to vehicle-only-treated control mice i.e., the relative expression of *Nrf2* mRNA in vehicle-only-treated control mice was 1.066 ± 0.169 while that of paclitaxel-treated mice was 0.761 ± 0.080. Interestingly, coadministration of paclitaxel with pristimerin significantly upregulated the expression of *Nrf2* mRNA compared to vehicle-only-treated control mice and paclitaxel plus vehicle-treated mice (Figure 7, *p* < 0.05).

## Discussion

This study shows all the four triterpenes (betulinic acid, cucurbitacin B, euphol and pristimerin) evaluated inhibited human recombinant MAGL activity, but only pristimerin did so in a concentration dependent manner. Pristimerin inhibited MAGL activity in mouse brain and paw skin tissues in a concentration dependent manner. Paclitaxel-induced mechanical allodynia was associated with increased MAGL activity in the paw skin. Treatment with pristimerin prevented the development of paclitaxel-induced mechanical allodynia and reduced the paclitaxel-induced increase in MAGL activity in the paw skin.

The first characterized MAGL inhibitors of plant origin are the triterpenes pristimerin and euphol using purified rat recombinant MAGL, with high IC_50_s of 93 and 315 nM, respectively (King et al., 2009). In the current study, pristimerin inhibited human recombinant MAGL with IC_50_ of 130 nM, which was comparable to the values obtained by King et al. (2009) utilizing purified rat recombinant MAGL (King et al., 2009) and Chicca et al. utilizing human recombinant MAGL with IC_50_ of 204 nM (Chicca et al., 2012). On the other hand, although euphol inhibited human recombinant MAGL its effects were not concentration dependent and thus could not calculate IC_50_ values. We did not find any studies that evaluated the effects of euphol on human recombinant MAGL activity. Betulinic acid and cucurbitacin B, similar to euphol, inhibited MAGL activity but their effects were not concentration dependent. It was reported that betulinic acid “did not to hit,” i.e., did not significantly inhibit, human MAGL although it inhibited human MAGL activity by about 23% at a concentration of 10 µM (Parkkari et al., 2014). In the current study betulinic acid at a concentration of 10 µM significantly inhibited human recombinant MAGL activity by about 35%. We did not find any studies that evaluated the effects of cucurbitacin B on human recombinant MAGL activity, however it effects on human recombinant MAGL was almost similar to that of betulinic acid. The magnitude of human recombinant MAGL inhibition by 1 µM of each of the triterpenes was pristimerin (50% inhibition) > betulinic acid (30% inhibition) ≥ cucurbitacin B (25% inhibition) > euphol (16% inhibition). Since pristimerin had a clear inhibitory effect on human recombinant MAGL, we evaluated its effects on mouse brain and paw skin MAGL activity. Pristimerin inhibited both mouse brain and paw skin MAGL activity in a concentration-dependent manner with IC_50_s of 1.6 µM in the brain and 0.596 µM in the paw skin, respectively. The differences in the potencies of pristimerin between the human recombinant MAGL and the mouse tissues MAGL could be because of competing reactions, similar to what was described by King et al. (2009) because of the presence of other enzymes that pristimerin might bind to in the tissues.

In a recent study we observed that there was a decrease in the amount of 2-AG in the paw skin of mice with paclitaxel-induced mechanical allodynia (Thomas et al., 2020). However, the protein levels of MAGL, the enzyme that degrades 2-AG, were not significantly altered (Thomas et al., 2020). In the current study we found that mice treated with paclitaxel had increased MAGL activity in the paw skin compared to vehicle-treated animal. Thus, the increased MAGL activity most likely resulted in the decreased 2-AG level, which contributed to the paclitaxel-induced mechanical allodynia. Indeed, replacement of 2-AG by local administration of 2-AG or the MAGL inhibitor JZL184 alleviated paclitaxel-induced mechanical allodynia (Thomas et al., 2020). In other studies, systemic administration of MAGL inhibitors JZL184 and MJN110 reversed paclitaxel-induced allodynia (Curry et al., 2018; Slivicki et al., 2018). Thus, it was plausible that pristimerin, a MAGL inhibitor (King et al., 2009) could prevent the development of paclitaxel-induced allodynia. Indeed, treatment with pristimerin prevented the development of paclitaxel-induced allodynia and reduced the paclitaxel-induced increase in MAGL activity. Compared to other MAGL inhibitors such as JZL184 and MJN110, which did not alter the anticancer activities of paclitaxel (Curry et al., 2018), pristimerin might have an advantage because it had an additive effects on paclitaxel’s anticancer activities against breast cancer cell lines (Lee et al., 2018). Thus, co-treatment with paclitaxel could have a dual advantage of reducing the dose of paclitaxel, PINP is a dose-dependent side effect, and also directly preventing PINP. This study evaluated the effects of pristimerin only on paclitaxel-induced mechanical allodynia, however, cold sensitivity is a pronounced side effect in CINP patients induced more by the platinum chemotherapeutic agents (Maihofner et al., 2021), thus further studies are needed to evaluate pristimerin effects on chemotherapy-induced cold hypersensitivity.

Oxidative stress and loss of epidermal nerve fibers are some of the major mechanisms involved in the pathogenesis of CINP (Han and Smith, 2013; Burgess et al., 2021). One of the major transcription factors involved in the regulation antioxidant response element–dependent genes is Nrf2 (Raghunath et al., 2018; He et al., 2020). It upregulates the expression of genes of molecules that protects against oxidative stress induced by xenobiotics and other stressors (Raghunath et al., 2018; He et al., 2020). Animals with paclitaxel-induced neuropathy and mechanical hypersensitivity had decreased expression of Nrf2 protein in the dorsal root ganglions (Zhao et al., 2019; Kumar Kalvala et al., 2022), which was reversed by treatment with drugs that have antihyperalgesic activity such as the cannabinoids cannabidiol and tetrahydrocannabivarin (Kumar Kalvala et al., 2022). In the current study, paclitaxel caused a tendency towards a decrease of *Nrf2* mRNA, and coadministration of pristimerin with paclitaxel significantly upregulated *Nrf2* mRNA. This suggests that pristimerin might prevent PINP also through upregulation of Nrf2 and having antioxidant activities. Further studies are warranted to explore whether pristimerin protects against PINP *via* upregulation and restoration of the antioxidant activities in tissues exposed to paclitaxel.

In conclusion the results of this study show that the triterpene pristimerin inhibits human recombinant MAGL activity as well as MAGL activity in mouse brain and paw skin tissues. Paclitaxel-induced mechanical allodynia is associated with increased MAGL activity in the paw skin, which most likely contributed to reduced 2-AG levels reported previously in animals with paclitaxel-induced mechanical allodynia (Thomas et al., 2020). Treatment with pristimerin, a triterpene with MAGL inhibitory activity prevented the development of paclitaxel-induced mechanical allodynia and reduced the paclitaxel-induced increase in MAGL activity, while increasing the expression of Nrf2, which controls antioxidant response element–dependent genes expression to protect against oxidative stress damage. Pristimerin has also been shown to have additive anticancer activity with paclitaxel against breast cancer cell lines (Lee et al., 2018). This could lead to lower doses of paclitaxel being used for treating cancer and thus reduce the occurrence of the dose dependent PINP. Therefore, pristimerin, and possibly other triterpenes, warrants further research as a potential candidate to be used in combination with paclitaxel for treatment of cancer and to prevent the development of PINP.

## Data Availability

The raw data supporting the conclusions of this article will be made available by the authors, without undue reservation.
